# Management of Clinically Involved Lateral Lymph Node Metastasis in Locally Advanced Rectal Cancer: A Radiation Dose Escalation Study

**DOI:** 10.3389/fonc.2021.674253

**Published:** 2021-07-16

**Authors:** Xiaolin Pang, Liang Huang, Yan Ma, Zhanzhen Liu, Peiyi Xie, Hailing Liu, Xiangbo Wan, Shuai Liu, Jian Zheng

**Affiliations:** ^1^ Department of Radiation Oncology, The Sixth Affiliated Hospital of Sun Yat-sen University, Guangzhou, China; ^2^ Guangdong Provincial Key Laboratory of Colorectal and Pelvic Floor Diseases, The Sixth Affiliated Hospital, Sun Yat-Sen University, Guangzhou, China; ^3^ Department of Colorectal Surgery, the Sixth Affiliated Hospital of Sun Yat-sen University, Guangzhou, China; ^4^ Guangdong Institute of Gastroenterology, The Sixth Affiliated Hospital of Sun Yat-sen University, Guangzhou, China; ^5^ Department of Radiology, the Sixth Affiliated Hospital of Sun Yat-sen University, Guangzhou, China; ^6^ Department of Pathology, the Sixth Affiliated Hospital of Sun Yat-sen University, Guangzhou, China

**Keywords:** locally advanced rectal cancer, lateral lymph node, lateral lymph node dissection, dose escalation, MRI

## Abstract

**Background:**

Patients with lateral lymph nodes (LLNs) metastasis are not effectively treated with neoadjuvant chemoradiotherapy. This study aimed to compare the efficacy of three neoadjuvant therapeutic regimens, namely, chemotherapy, chemoradiotherapy, and chemoradiotherapy with a dose boost of LLNs, and to identify the optimal approach for treating LLNs metastasis of locally advanced rectal cancer.

**Methods:**

A total of 202 patients with baseline LLNs metastasis (short axis ≥5 mm) and treated with neoadjuvant treatment, followed by radical surgery from 2011 to 2019, were enrolled. The short axis of the LLNs on baseline and restaging MRI were recorded. Survival outcomes were compared.

**Results:**

In the booster subgroup, shrinkage of LLNs was significantly greater than in the neoadjuvant chemotherapy and chemoradiotherapy subgroups (*P <*0.001), without increasing radiation related side effects (*P =* 0.121). For patients with baseline LLNs of short axis ≥5 mm in the booster subgroup, the response rate (short axis <5 mm on restaging MRI) was 72.9%, significantly higher than patients in the neoadjuvant chemotherapy subgroup (48.9%, *P* = 0.007) and higher than for patients in the neoadjuvant chemoradiotherapy group (65.0%), but there was no statistical difference (*P* = 0.411). The 3-year local recurrence and lateral local recurrence rates were both 2.3% in the dose booster group, which were lower than those of the other two subgroups (local recurrence: *P <*0.001; lateral local recurrence: *P <*0.001). The short axis of lateral lymph nodes (≥5 and <5 mm) on restaging MRI was an independent risk factor for prognosis (*P <*0.05).

**Conclusion:**

Radiation dose boost is an effective way of increasing the response rate and decreasing recurrence rates. The restaging LLNs with short axis ≥5 mm is a predictor of poor prognosis.

## Introduction

Neoadjuvant chemoradiotherapy (nCRT), followed by total mesorectal excision (TME), has become the standard treatment for locally advanced rectal cancer (LARC) (1). However, 5–8% of patients continue to experience local recurrence after 3 years (1–3). Previous studies have confirmed that lateral lymph node (LLN) metastasis is one of the most important factors influencing recurrence in middle and low rectal cancer (4, 5). In some Asian countries, lateral lymph node dissection (LLND) is recommended for patients with LARC (6, 7). However, LLND is associated with various complications, including longer operation time, larger blood loss, and severe sexual and urinary dysfunction (7, 8). In contrast, in western countries, LLND is not performed regularly, and nCRT before TME surgery is considered standard treatment (9).

Numerous studies have suggested that preoperative nCRT does not eradicate LLN metastasis, especially when the short axis (SA) is persistently greater than 5 mm after standard nCRT, and the pathologically positive rate was observed in 60–75% of cases (10–12). In contrast, when the LLNs show a favorable response (SA <5mm) to nCRT, the positive rate is reduced to <20% (12). Therefore, to avoid overtreatment and morbidity, it is strongly recommended that LLND should be delivered to patients with LLNs who do not respond well to nCRT (restaging SA ≥5 mm) (10, 13, 14). However, it has been reported that after standard 45 Gy radiation of LLNs, the response rate of the LLNs was in the range of 45.5–56.1% (4, 12, 15).

Considering the development of TME surgery and the side effect of radiotherapy, a strategy of removing neoadjuvant radiotherapy by intensified neoadjuvant chemotherapy was proposed for LARC. Prospective trials, such as a phase II study and FORWAC, revealed that the intensified nCT treatment without radiotherapy might be a promising way to improve oncological outcomes for LARC (2, 16). However, for LARC patients with LLN metastasis, the effect of omitting neoadjuvant radiotherapy by intensifying the neoadjuvant chemotherapy was unclear.

Radiation dose escalation studies have shown that increasing radiation doses could improve local control (17, 18). In recent years, the simultaneous integrated boost intensity-modulated radiotherapy treatment (SIB-IMRT) strategy has been implemented, allowing the simultaneous delivery of various dose prescriptions and target volumes in the same fraction, thus avoiding a delay in total treatment time (18). In a prospective study, SIB-IMRT has been shown to improve the pathological complete response rate with acceptable toxicity effects for patients with LARC (17). However, the findings of studies on the escalation of LLNs in rectal cancer are unclear. There was only one single-center study (12 cases) that tried to boost the LLN doses to 60 Gy, while long-term follow-up was not reported (19).

In this large-scale retrospective cohort study, we compared three different treatment regimens: neoadjuvant chemotherapy (nCT), nCRT, and neoadjuvant chemoradiotherapy with a radiation dose boost (nCRT-boost) of LLNs. The aim was to identify the optimal neoadjuvant treatment regimen of LLNs metastasis in patients with LARC. This study was approved by the central ethics committee of the Sixth Affiliated Hospital, Sun Yat-sen University (No. 2020ZSLYEC-274).

## Methods

### Study Population

This was a retrospective study that included patients from January 2011 to October 2019 at a gastrointestinal specialist hospital. Patients who were staged clinically T3–T4 and N-positive for rectal adenocarcinoma were included. Other inclusion criteria were as follows: adenocarcinoma was located within 10 cm of the anal verge; the patient had undergone neoadjuvant chemoradiotherapy or chemotherapy, followed by TME surgery; the baseline and restaging MRI scans were available; there was at least one baseline LLN (SA ≥5 mm) at the internal iliac, obturator, and external iliac region. The exclusion criteria included the presence of distant metastases at diagnosis or before TME surgery, the absence of MRI scans, and/or poor scan quality.

### Radiotherapy

Patients received 5-field SIB-IMRT with an Elekta Synergy accelerator. From July 2015, an attempt at radiation dose escalation of baseline LLNs (SA ≥5 mm) was made. The gross tumor volume (GTV) was defined as gross disease determined from MRI scans. The lymph nodes (SA ≥5 mm) at the internal iliac, obturator, and external iliac regions were delineated, named GTVnd, and were given a radiation dose boost. The clinical target volume (CTV) was defined as the GTV and GTVnd plus areas considered at significant risk of harboring microscopic area. The planning target volume (PTV) was generated by adding an 8-mm margin around the GTV, GTVnd, and CTV in all directions. Doses of 56–58, 50, and 45 Gy were delivered to PTV-GTVnd, PTV-GTV, and PTV-CTV at 25 fractions, respectively. The dose of the normal organs at risk was based on the following criteria: bowel bag, V50 ≤5%; bladder, V50 ≤50%; femoral heads, V50 ≤5% (20).

### Chemotherapy

During radiotherapy treatment, intravenous fluoropyrimidine-based chemotherapy was concurrently administered. Patients were given fluoropyrimidine-based consolidation chemotherapy during the waiting time before TME surgery. The regimens were fluorouracil-based, consisting of fluorouracil; folinic acid, fluorouracil, and oxaliplatin (FOLFOX); or irinotecan and fluorouracil (FOLFIRI). A subgroup of patients enrolled in a prospective study did not receive neoadjuvant radiotherapy (2). After TME surgery, fluoropyrimidine-based chemotherapy was administered. Four to six cycles of chemotherapy were administered before surgery, and six to eight cycles were administered as postoperative adjuvant chemotherapy (2).

### Surgical Procedure

Surgery with curative intent was performed according to TME principles at six to eight weeks following the completion of neoadjuvant radiotherapy or two weeks after completion of neoadjuvant chemotherapy (2, 21). Surgery was performed by the experienced director, who had been trained for at least 10 years in a third-grade class A hospital.

### Evaluation of MRIs

Two experienced physicians reviewed the baseline and restaging MRI scans. The baseline MRI examination was performed within two weeks before the beginning of treatment; LLNs in the internal iliac, obturator, or external iliac regions were recorded when SA was ≥5 mm with or without morphological changes. After neoadjuvant treatment, the reduction in SA size on the restaging MRI scans before the TME surgery was also recorded. The examination time of the restaging MRI was within two weeks after the consolidation chemotherapy and one week before the TME surgery. When the size of SA was <5 mm on the restaging MRI scan, it was defined as LLN-responsive. When the two experts came to different conclusions, a third physician would make the final decision.

### Follow-up

After the TME surgery, all patients were followed up at three-month intervals during the first three years and thereafter at six-month intervals. Physical examinations, chest and abdomen CT, and contrast-enhanced pelvic MRI would be monitored. Lateral local recurrence (LLR) was defined as recurrence at internal iliac, obturator, and external iliac lymph nodes sites, and local recurrence (LR) was defined as recurrence at one of five sites—lateral, presacral, anastomotic site, anterior, or perineal. Distant recurrence (DR) was defined as distant recurrence, when censored, at the latest time. Cancer-specific survival (CSS) was defined as time from the date of surgery to death caused by tumor progression or, when censored, at the latest date if the patient was still alive.

### Statistical Analyses

Statistical analyses were performed using IBM SPSS v26 (Chicago, IL, USA). Individual variables were compared by *t*-tests. Survival curves for LR, LLR, DR, and CSS were calculated using the Kaplan–Meier method. To compare the degrees of the reduction of SA of LLN in different treatments, ANOVA was used. To identify risk factors, a univariate Cox regression model was employed, and for patients with multiple LLNs on primary MRI scan, only the largest LLN was analyzed. Differences with a *p*-value of 0.05 were considered statistically significant.

## Results

### Patient Characteristics

**Figure 1:**
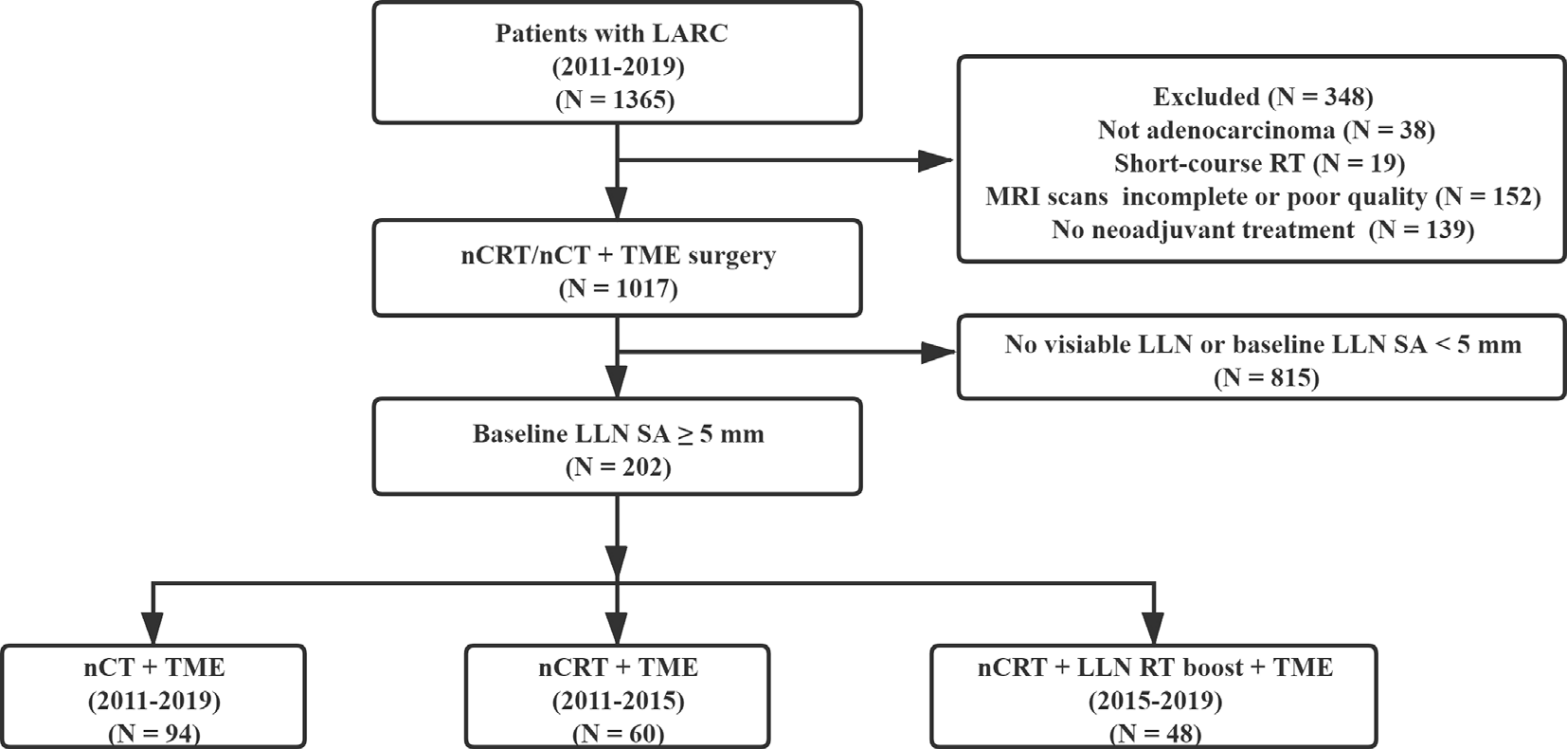
Patient flowchart.

In this study, at least one LLN with SA ≥5 mm was detected in the baseline MRI in 202 cases (**Figure 1**). The median time interval between restaging MRI and surgery was five days (IQR, 0–7 days). Among the 202 patients with clinically positive LLNs on baseline MRI, 94 cases (46.5%) were treated by nCT, 60 cases (29.7%) were treated by nCRT, and 48 cases (23.8%) were given nCRT-boost therapy. Except for the distribution of the LLNs (unilateral or bilateral), there were no statistically significant differences in other variables among the three subgroups (all *P >*0.05). In the nCRT-boost subgroup, there were more patients with bilateral metastasis LLNs than those in the nCT and nCRT subgroups (*P* = 0.012) (**Table 1**).

**Table 1:** Clinicopathological characteristics of 202 patients with LLN metastasis.

Variables	nCT No. (%) n = 94	nCRT No. (%) n = 60	nCRT-boost No. (%) n = 48	*P*-value
Age, median 55 years				0.897
<55	45 (47.9)	31 (51.7)	24 (50.0)	
≥55	49 (52.1)	29 (48.3)	24 (50.0)	
Gender				0.382
Male	64 (68.1)	47 (78.3)	34 (70.8)	
Female	30 (31.9)	13 (21.7)	14 (29.2)	
Clinical T stage				0.320
cT2	1 (1.1)	3 (5.0)	2 (4.2)	
cT3–4	93 (98.9)	57 (95.0)	46 (95.8)	
Clinical N stage				0.287
cN1	32 (34.0)	28 (46.7)	18 (37.5)	
cN2	62 (66.0)	32 (53.3)	30 (62.5)	
Location from anal verge (cm)				0.058
0–5	50 (53.2)	41 (68.3)	34 (70.8)	
5–10	44 (46.8)	19 (31.7)	14 (29.2)	
Tumor differentiation				0.864
Highly differentiated	26 (27.7)	19 (31.7)	16 (33.3)	
Moderately differentiated	54 (57.4)	31 (51.7)	23 (47.9)	
Poorly differentiated	14 (14.9)	10 (16.6)	9 (18.8)	
LLN metastasis				**0.012**
Unilateral	89 (94.7)	55 (91.7)	38 (79.2)	
bilateral	5 (5.3)	5 (8.3)	10 (20.8)	
Chemotherapy regimen				0.947
5-Fu	30 (31.9)	20 (33.3)	15 (31.3)	
FOLFOX	55 (58.5)	36 (60.0)	30 (62.5)	
FOLFIRI	9 (9.6)	4 (6.7)	3 (6.2)	
Location				0.207
Inter iliac	45 (48.4)	40 (66.7)	24 (50.0)	
Obturator	37 (39.8)	15 (25.0)	20 (41.7)	
External iliac	12 (11.8)	5 (8.3)	4 (8.3)	
Surgery type				0.760
Sphincter-preserving operation	85 (90.4)	52 (86.7)	43 (89.6)	
Abdominoperineal resection	9 (9.6)	8 (13.3)	5 (10.4)	

The bold type indicates that the P value is statistically significant.

All of the patients underwent TME surgery without LLND, and no patients were surgically margin positive. After the radical surgery, there were no statistically significant differences in the pathologic N stage after neoadjuvant therapy and TME surgery (ypN), vascular and neural invasion, circumferential resection margin (CRM), and adjuvant chemotherapy treatment among the three subgroups (all *P >*0.05). However, patients in the nCT subgroup had more advanced pathologic T stage after neoadjuvant therapy and TME surgery (ypT) (*P <*0.001) and the American Joint Committee on Cancer and College of American Pathologists Tumor Regression Grade (AJCC/CAP TRG) stages (*P <*0.001) (**Supplementary Table 1**).

### Primary and Restaged MRI Scans

Based on the largest SA of LLNs on the baseline MRI scans, the mean SA of the LLNs was 8.0 mm (IQR, 5.0–20.3 mm), 8.2 mm (IQR, 5.0–58.0 mm), and 9.6 mm (IQR, 5.2–41.0 mm) in nCT, nCRT, and nCRT-boost subgroups, respectively. After the neoadjuvant treatment, size shrinkage in the nCRT-boost subgroup was greater than that in the nCT and nCRT subgroups (nCRT-boost vs. nCT, *P <*0.001; nCRT-boost vs. nCRT, *P* = 0.030). On the restaging MRI, 120 cases (59.4%) with reduced LLNs were smaller than 5 mm. In the nCRT-boost subgroup, the response rate of the internal iliac nodes, obertuotor nodes, and external iliac nodes were 62.5% (15/24), 95.0% (19/20), and 25.0% (1/4), respectively (internal iliac nodes vs. obertuotor nodes, *P =* 0.013; internal iliac nodes vs. external iliac nodes, *P =* 0.285). The entire response rate of the patients in the nCRT-boost subgroup was 72.9% (35/48), which was significantly higher than those of the patients in the nCT (46/94, 48.9%, *P* = 0.007). The entire response rate of the patients in the nCRT-boost group was also higher than those in the nCRT group (39/60, 65.0%), but there was no statistical difference (*P* = 0.411). (**Table 2** and **Supplementary Table 2**, **Supplementary Figure 1**).

**Table 2:** Baseline and restaging MRI values of LLN SA for patients treated by three different treatment regimens (n = 202).

Variable	nCT n = 94	nCRT n = 60	nCRT-boost n = 48	*P-*value
Baseline SA mean, (mm)	8.0 (5.0–20.3)	8.2 (5.0–58.0)	9.6 (5.2–41.0)	0.208
Shrinkage SA mean, (mm)	3.2 (−1.9–10.7)	4.9 (−0.4–46.0)	6.6 (0.2–16.6)	**<0.001**
Restaging SA mean, (mm)	4.7 (0–18.0)	3.3 (0–14.0)	3.0 (0–24.4)	**0.016**
Response rate (%) (Restaging SA <5 mm)	48.9% (46/94)	65.0% (39/60)	72.9% (35/48)	**0.013**

The bold type indicates that the P value is statistically significant.

For further analysis of the pathological characters of patients with restaging LLNs in SA ≥5 mm or SA <5 mm, there were significantly more advanced ypT, ypN, and AJCC/CAP TRG scales in patients with restaging LLN in SA ≥5 mm (all *P <*0.05). Especially, a higher number of patients in the subgroup with SA ≥5 mm received adjuvant treatment than those with SA <5 mm (*P* = 0.003) (**Supplementary Table 3**).

### Side Effects of Escalation Radiotherapy of LLNs

In the nCRT and nCRT-boost groups, all patients completed radiotherapy. In the nCRT-boost group, a median of 58 Gy (IQR, 56–58 Gy) was boosted in 58 lateral lymph nodes of 48 patients (56 Gy: 23 LLNs; 58 Gy: 35 LLNs). Using the criteria of CTCAE v4.0 (22), the occurrence rate of radiation-related grades 3–4 complications was 29.1% in the nCRT-boost group, and a comparison of the occurrence of toxicity of the patients in the nCRT group did not reveal any significant differences (*P* = 0.121**)** (**Supplementary Table 4**).

### Survival Analysis

**Figure 2:**
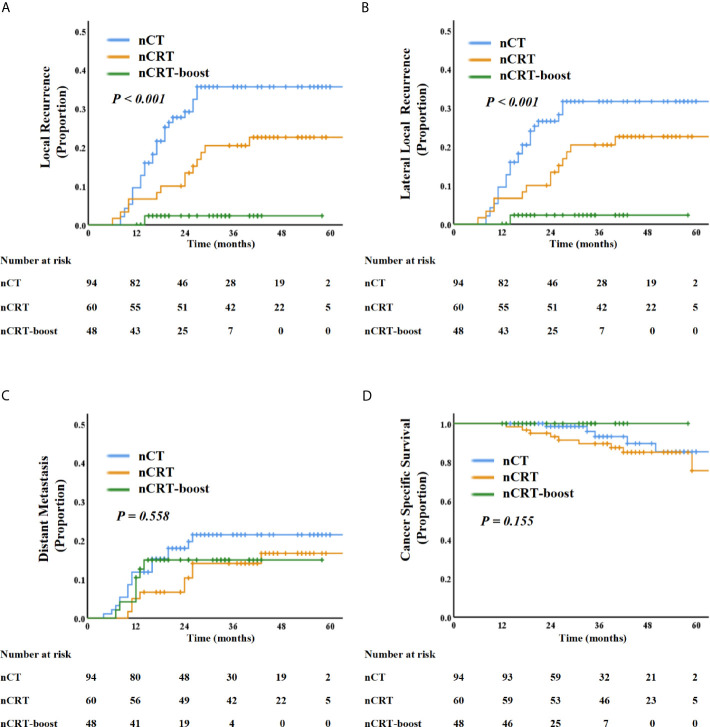
Kaplan–Meier curve analysis of local recurrence **(A)**, lateral local recurrence **(B)**, distant recurrence **(C)**, and cancer-specific survival **(D)** comparing the patients with LLNs metastasis underwent three different treatment regimens: nCT, nCRT, and nCRT-boost treatment.

For the 202 patients, the median follow-up time was 35 months (IQR: 12.0–82.0 months). LR was observed in 44 patients, including 30 cases (68.2%) in nCT subgroup, 13 cases (29.5%) in the nCRT subgroup, and only one case (2.3%) in the nCRT-boost subgroup. Out of the 44 patients with LR, 30 had primary internal iliac nodes metastasis (68.2%, 30/44), 14 had primary obturator nodes metastasis (31.8%, 14/44), but none had primary external iliac nodes metastasis (0%, 0/44). Furthermore, 41 local recurrence cases out of the total 44 (93.2%) developed LLR, and 20 patients (45.5%) developed distant metastasis. The LR rates for different cut-off values in SA in baseline LLN-positive clinical patients who received neoadjuvant treatment are listed in **Table 3**. For patients with baseline LLNs SA ≥5 mm, the 3-year LR rate reached 25.1%. We then compared three different neoadjuvant treatment regimens on those patients; the 3-year LR and LLR rates in the nCRT-boost subgroup were lower than the nCT and nCRT subgroups (LR, 2.3% *vs*. 35.6% *vs.*20.4%, *P <*0.001; LLR, 2.3% *vs*. 31.6% *vs.* 20.4%, *P <*0.001) (**Figure 2**).

**Table 3:** Three-year local recurrence rates for different cutoff values in SA on baseline MRI in patients with lateral node metastasis.

SA, mm	No. (%)	3-year LR (%)	*P*-value
SA 5			*/*
<5	*/*	*/*	
≥5	202 (100.0)	25.1	
SA 6			**0.002**
<6	58 (28.7)	9.3	
≥6	144 (71.3)	32.1	
SA 7			**<0.001**
<7	79 (39.1)	8.6	
≥7	123 (60.9)	34.1	
SA 8			**0.001**
<8	117 (57.9)	14.3	
≥8	85 (42.1)	36.2	
SA 9			**<0.001**
<9	141 (69.8)	14.5	
≥9	61 (30.2)	45.4	
SA 10			**<0.001**
<10	153 (75.7)	14.2	
≥10	49 (24.3)	52.6	

The bold type indicates that the P value is statistically significant.

**Figure 3:**
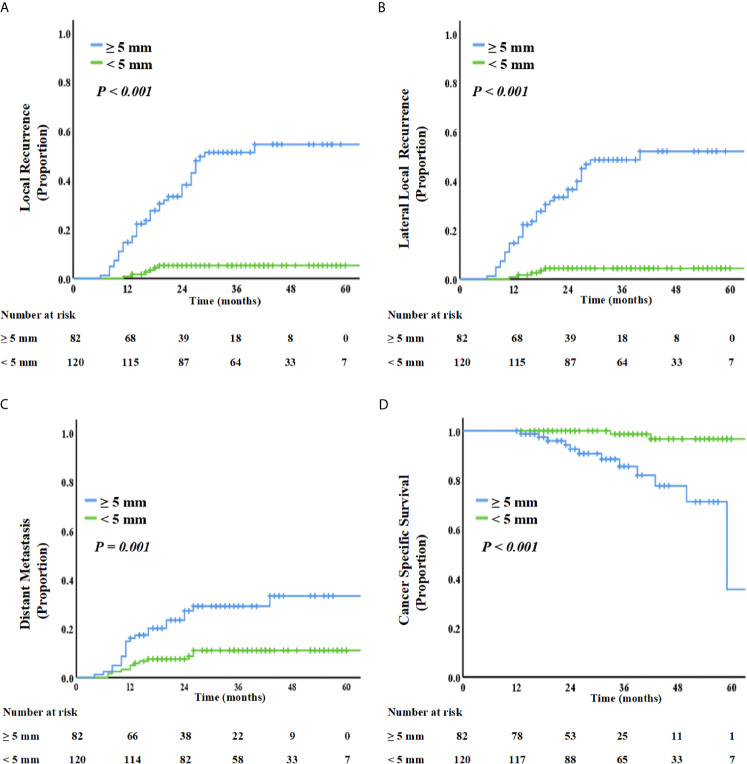
Kaplan–Meier curve analysis of local recurrence **(A)**, lateral local recurrence **(B)**, distant recurrence **(C)**, and cancer-specific survival **(D)**, comparing patients with LLN SA ≥5 mm or <5 mm on restaging MRI.

On restaging MRI scans, 120 patients with LLNs disappeared or with SA <5 mm; however, 82 patients were persistently with LLNs ≥5 mm. As summarized in **Figure 3**, the SA of LLNs (≥5 mm vs. <5 mm) was a significant influencing factor for 3-year LR, LLR, DR, and OS. Patients with LLNs ≥5 mm in SA had a significantly high LR (51.3% *vs*. 5.3%, *P <*0.001), LLR (48.6% *vs*. 4.4%, *P <*0.001), DR (29.2% *vs*.11.1%, *P =* 0.001), and poor CSS (85.5% *vs*. 98.6%, *P <*0.001), compared with those who had LLNs <5 mm in SA.

Furthermore, the associations between the restaging SA size, LR, and LLR rates were analyzed. The SA cut-off value of the LLNs on restaging MRI was 5 mm. The areas under the ROC curve (AUC) for LR and LLR were 0.903 and 0.906, respectively (**Supplementary Figure 2**).

### Univariable and Multivariable Analyses

As summarized in **Supplementary Tables 5** and **6**, patients with restaging LLNs ≥5 mm in SA had a significantly higher risk of LR (HR, 8.880; 95% CI, 3.660 to 21.544; *P <*0.001), LLR (HR, 11.992; 95% CI, 4.679 to 30.731; *P <*0.001), DR (HR, 2.118; 95% CI, 1.006 to 4.460; *P* = 0.048), and poor CSS (HR, 8.456; 95% CI, 1.766 to 40.495; *P* = 0.008) than patients with LLNs <5 mm in SA. In addition, compared with patients in the nCT and nCRT groups, patients treated with nCRT-boost showed independent prognosticators of 3-year LR (HR, 0.075; 95% CI, 0.010 to 0.552; *P* = 0.011).

## Discussion

In our study, we evaluated the survival outcomes in patients with LLN clinical metastasis who underwent three different neoadjuvant treatments. Our study showed that for patients with baseline LLNs SA ≥5 mm, the nCRT-boost to LLNs decreased the LR and LLR rates and reduced the SA of LLNs compared to nCT and nCRT treatments. In addition, the response rate of LLNs in the nCRT-boost group was significantly higher than the response rate of LLNs in the nCT group and tended to be higher than that in the nCRT group. After neoadjuvant treatment, restaging LLNs SA ≥5 mm was associated with inferior LR, LLR, DR, and poor CSS.

According to previous studies, baseline LLNs SA cut-off from 5 to 10 mm were adopted as the clinically positive standard before nCRT (13, 23–25). In a recent large-scale study conducted by the Lateral Node Study Consortium, a baseline SA of 7 mm was adopted by the cut-off value, and 20% LLR rate was observed in those patients. However, for the patients with baseline LLN SA >5 mm in that study, the LLR rate was also approximately 16% (23). Indeed, LLN SA >5 mm on MRI scans has been proposed as the best cut-off standard in several studies (10, 13, 14). A multicenter MRI study showed that when 5 mm was used as the cut-off value, the LR rate was 21.7% (10). A cut-off value of 5 mm was selected for our study, and the LR rate was 25.1%.

There was a need for a consensus in selecting LLND for the patients based on the restaging imaging findings of LLNs (10, 12, 13, 25, 26). A study conducted at the MD Anderson Cancer Center found that after nCRT, none of the patients with LLN <5 mm had pathologically positive LLNs (13). Oh et al. analyzed 66 patients with suspected LLN involvement who underwent nCRT and LLND, and none of the LLNs were pathologically positive for SA <5 mm; especially for the restaging LLN SA ≥5 mm, the LR rate reached 45.4% (12). Thus, several studies have confirmed that LLN SA ≥5 mm is the optimal criterion for selecting patients for LLND, which might reduce the LR rate, and a recent study has also shown that LLND is also an effective way to decrease the relapse-free survival rate (10, 12, 25). Similarly, in our study, the 3-year LR was 51.3% for patients with restaging LLN SA ≥5 mm, although a greater proportion of patients received adjuvant chemotherapy than those with restaging LLN SA <5 mm. Moreover, our study showed that restaging LLNs with SA ≥5 mm was a significant and independent predictor of LR, LLR, DR, and CSS. The reason might be that the patients with poor reduction in LLN also showed poor tumor response to neoadjuvant treatment. This was demonstrated in our study, i.e., patients with restaging LLN SA ≥5 mm had more advanced ypT stages, ypN stages, and AJCC/CAP TRG stages. Thus, although none of the patients in our study underwent LLND, in light of the high LR rates for the patients with restaging LLN SA ≥5 mm, LLND might be employed.

Although minimally invasive surgery methods such as laparoscopy and robotics cause less bleeding and offer good nerve protection than open surgery (27), it is highly dependent on the experience of the surgeon and is thus generally difficult to perform. Therefore, how to increase the response rate of baseline LLN during the nCRT period is a major challenge. Here, an attempt at an escalation radiation dose based on the regular dose of 56–58 Gy was prescribed to a subgroup of patients with baseline LLN SA ≥5 mm from 2015. In line with our study, a radiation dose boost for the clinical suspicious LLNs was reported in several studies (19, 28, 29). In gynecologic cancers, the radiation dose of LLN was boosted to 60 Gy and did not result in higher morbidity rates (28). In the REG001-09 trial, a median dose of 66.5 Gy was given to the clinically involved prostate cancer lymph node, and the side effect of radiation was acceptable (29). To date, reports on LLN dose escalation in rectal cancer are limited. Only a small-scale study (involving 12 patients) with short-term LR was reported (19). In our study, for patients with LLN metastasis, the LR and LLR rates in the dose escalation subgroup were significantly lower, and size reduction was significantly better than in the nCT and nCRT subgroups. Especially, for patients with LLN SA ≥5 mm on baseline MRI, the response rate in the nCT subgroup was only 48.9%, indicating that the omitting radiotherapy was unfeasible. However, the response rate in the nCRT-boost subgroup was 72.9%, meaning that more patients would avoid LLND. Especially, in line with the studies from the Lateral Node Study Consortium, patients with obturator nodes metastasis achieved a much higher response rate and a lower recurrence rate than those with internal iliac nodes metastasis (30). Significantly, compared with the nCRT subgroup, the rates of enteritis and dermatitis in the nCRT-boost subgroup were similar. These findings suggest that radiation dose escalation might be an effective and acceptable treatment selection for LARC patients with LLN metastasis.

Our study has some limitations. First, it was a retrospective single-hospital study and thus may suffer from selection bias. Some patients with clinically LLN metastasis could not have been included due to unavailable or poor-quality MRI scans. Second, although the patients’ baseline clinicopathological characters were not significantly different among the three neoadjuvant treatment schemes, the number of patients included in each subgroup was relatively small. Third, none of the 202 patients enrolled in our study underwent LLND surgery, thus the pathology of the LLNs was missing. Finally, for the patients in the nCRT-boost group, the median follow-up was 30.5 months (IQR: 12.0–58.0 months). Thus, the findings of this study need to be warranted by long-term follow-up and other prospective clinical trials. These limitations were unavoidable given the retrospective nature of this study. A prospective study on the escalation of LLNs is currently being conducted at our hospital.

## Conclusions

For patients with LARC with baseline LLNs SA ≥5 mm, dose escalation of LLNs may lead to a significantly lower rate of LR and LLR. In addition, SA of restaging LLNs was an independent influence factor for prognosis. Especially, for patients with LLN metastasis, dose escalation of LLNs is an effective and acceptable way to reduce the size of LLNs, and LR and LLR rates, and increase the response rate of LLNs, thus allowing more patients to avoid LLND.

## Data Availability Statement

The raw data supporting the conclusions of this article will be made available by the authors, without undue reservation.

## Ethics Statement

Written informed consent was obtained from the individual(s) for the publication of any potentially identifiable images or data included in this article.

## Author Contributions

JZ and SL were the principal investigators and designed the study. XP performed contouring, treatment planning, and statistical analysis. YM, LH, and ZL provided patient data. XP, HL, XW, and PX checked the data. YM, LH, ZL, JZ, and SL reviewed all data. All authors discussed the data. JZ and XP wrote the draft manuscript and performed subsequent revisions, which were reviewed by all other authors. All authors contributed to the article and approved the submitted version.

## Funding

The National Natural Science Foundation for Young Scholars of China (Grant No. 81703080) and The Sixth Affiliated Hospital of Sun Yat-Sen University Clinical Research 1010 Program [Grant No. 1010PY (2020)-09] supported this study.

## Conflict of Interest

The authors declare that the research was conducted in the absence of any commercial or financial relationships that could be construed as a potential conflict of interest.

## Supplementary Material

Supplementary Figure 1 The baseline and restaging LLNs SA underwent nCT, nCRT, and nCRT-boost treatment.

Supplementary Figure 2 ROC curve analysis of LLNs SA on restaging MRI to predict individual risk to local recurrence (LR) **(A)** and lateral local recurrence (LLR) **(B)**.

## Abbreviations

LLN, lateral lymph node; nCRT, neoadjuvant chemoradiotherapy; TME, total mesorectal excision; LARC, locally advanced rectal cancer; LLND, lateral lymph node dissection; SIB-IMRT, simultaneous integrated boost intensity-modulated radiotherapy treatment; nCT, neoadjuvant chemotherapy; nCRT-boost, neoadjuvant chemoradiotherapy with a radiation dose boost; LLR, lateral local recurrence; LR, local recurrence; DR, distant recurrence; CSS, cancer-specific survival; AJCC/CAP TRG, American Joint Commission on Cancer and College of American Pathologists Tumor Regression Grade; GTV, gross tumor volume; CTV, clinical target volume; PTV, planning target volume; CRM, circumferential resection margin; HR, hazard ratio; CI, confidence interval; NCCN, National Comprehensive Cancer Network.
