# Supplementary figures and images for: Management of Clinically Involved Lateral Lymph Node Metastasis in Locally Advanced Rectal Cancer: A Radiation Dose Escalation Study
Source: Front Oncol. 2021 Jul 16;11:674253. doi: 10.3389/fonc.2021.674253 (PMC8322741; doi:10.3389/fonc.2021.674253)

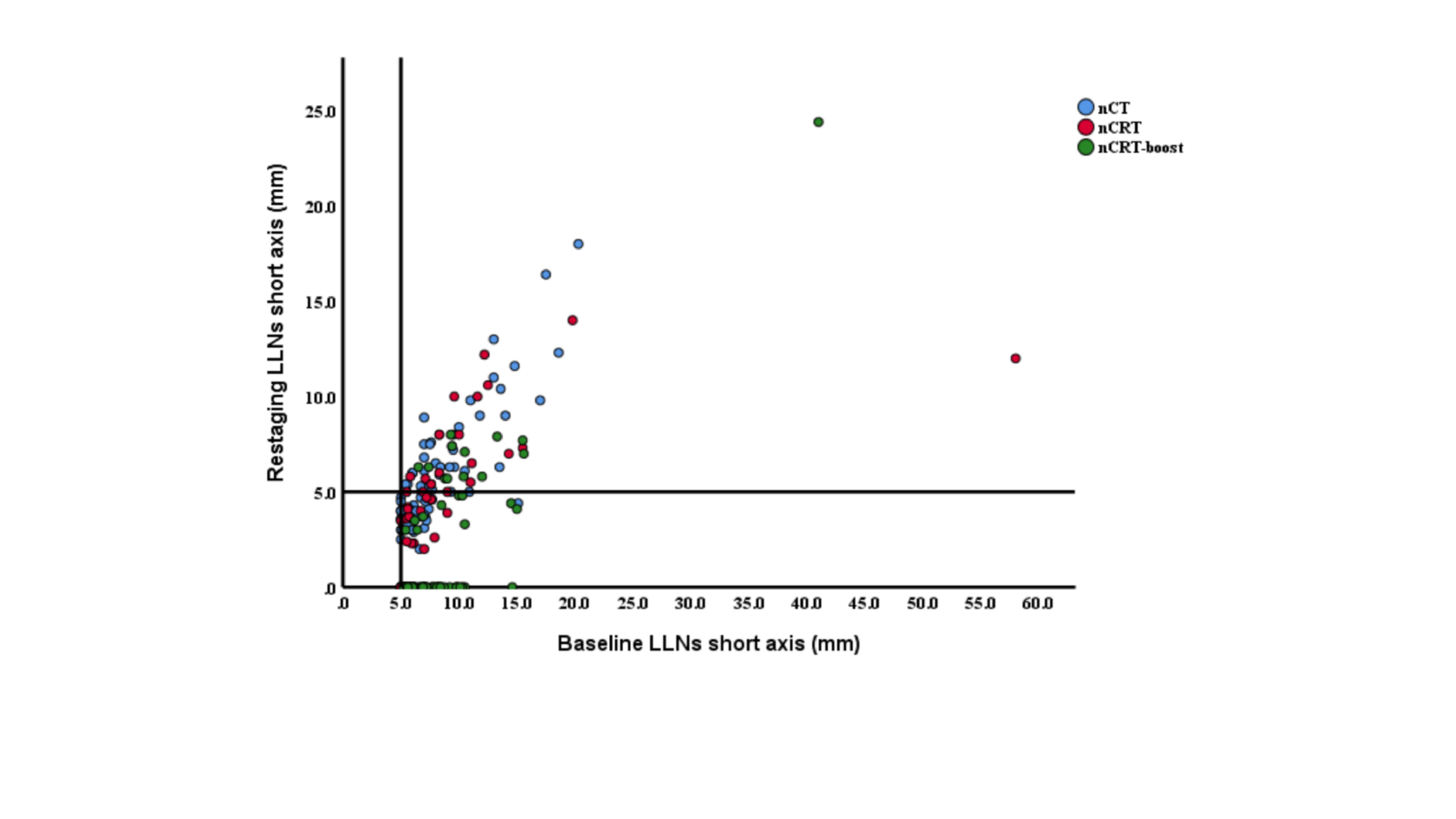

Supplement: Supplementary Figure 1 — The baseline and restaging LLNs SA underwent nCT, nCRT, and nCRT-boost treatment. [file Image_1.tif]

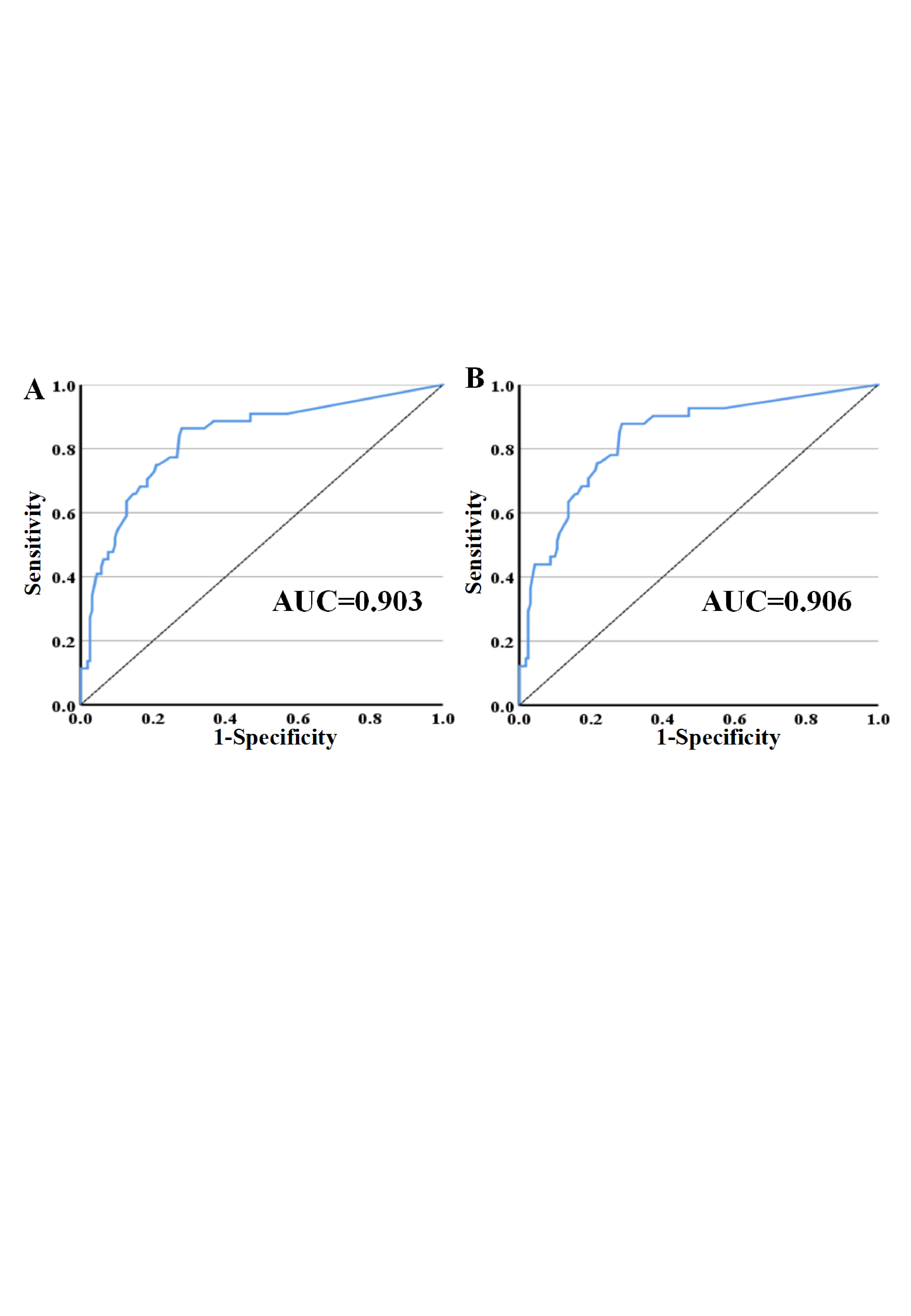

Supplement: Supplementary Figure 2 — ROC curve analysis of LLNs SA on restaging MRI to predict individual risk to local recurrence (LR) (A) and lateral local recurrence (LLR) (B). [file Image_2.tif]
