# Supplementary material for: Management of Clinically Involved Lateral Lymph Node Metastasis in Locally Advanced Rectal Cancer: A Radiation Dose Escalation Study
Source: Front Oncol. 2021 Jul 16;11:674253. doi: 10.3389/fonc.2021.674253 (PMC8322741; doi:10.3389/fonc.2021.674253)
Supplement: Supplementary file 3 [file Table_1.docx]

**SUPPLEMENTARY TABLE 1**. Patients clinicopathological characteristics with LLNs metastasis among three subgroups after neoadjuvant treatment and surgery (n = 202).

| **Variables** | **nCT** **No. (%)**  **n = 94** | **nCRT No. (%)**  **n = 60** | **nCRT-boost No. (%)**  **n = 48** | ***P*-value** |
| --- | --- | --- | --- | --- |
| ypT stage^a^ |  |  |  | **< 0.001** |
| yp T0 | 9 (9.6) | 25 (41.7) | 17 (35.4) |  |
| yp T1 | 9 (9.6) | 6 (10.0) | 4 (8.3) |  |
| yp T2 | 19 (20.2) | 6 (10.0) | 12 (25.0) |  |
| yp T3 | 52 (55.3) | 22 (36.7) | 15 (31.3) |  |
| yp T4 | 5 (5.3) | 1 (1.6) | 0 (0.0) |  |
| yp N stage^a^ |  |  |  | 0.174 |
| yp N0 | 66 (70.2) | 50 (83.3) | 37 (77.1) |  |
| yp N1-2 | 28 (29.8) | 10 (16.7) | 11 (22.9) |  |
| AJCC/CAP TRG |  |  |  | **< 0.001** |
| 0 | 9 (9.6) | 25 (41.7) | 17 (35.4) |  |
| 1 | 11 (11.7) | 15 (25.0) | 12 (25.0) |  |
| 2 | 49 (52.1) | 18 (30.0) | 19 (39.6) |  |
| 3 | 25 (26.6) | 2 (3.3) | 0 (0.0) |  |
| Vascular invasion |  |  |  | 0.163 |
| negative | 89 (94.7) | 59 (98.3) | 48 (100.0) |  |
| positive | 5 (5.3) | 1 (1.7) | 0 (0.0) |  |
| Neural invasion |  |  |  | 0.154 |
| negative | 87 (92.6) | 57 (95.0) | 48 (100.0) |  |
| positive | 7 (7.4) | 3 (5.0) | 0 (0.0) |  |
| Surgical margin |  |  |  | / |
| negative | 94 (100.0) | 60 (100.0) | 48 (100.0) |  |
| positive | 0 (0.0) | 0 (0.0) | 0 (0.0) |  |
| Circumferential resection margin, mm |  |  |  | 0.561 |
| ≤ 1 | 93 (98.9) | 60 (100.0) | 48 (100.0) |  |
| > 1 | 1 (1.1) | 0 (0.0) | 0 (0.0) |  |
| Adjuvant chemotherapy |  |  |  | 0.713 |
| Yes | 83 (88.3) | 52 (86.7) | 40 (83.3) |  |
| No | 11 (11.7) | 8 (13.3) | 8 (16.7) |  |

*^a^yp stage is pathological stage after neoadjuvant treatment and surgical resection.*

*The bold type indicates that the P value is statistically significant.*
