# Supplementary material for: Management of Clinically Involved Lateral Lymph Node Metastasis in Locally Advanced Rectal Cancer: A Radiation Dose Escalation Study
Source: Front Oncol. 2021 Jul 16;11:674253. doi: 10.3389/fonc.2021.674253 (PMC8322741; doi:10.3389/fonc.2021.674253)
Supplement: Supplementary file 4 [file Table_2.docx]

**SUPPLEMENTARY TABLE 2. Pairwise comparisons of the baseline and restaging MRI values of LLN SA for patients treated by three different treatment regimens (n = 202).**

| **Variable** |  | **nCRT** | **nCRT-boost** |
| --- | --- | --- | --- |
| Baseline SA mean (mm) | nCT | 0.814 | 0.085 |
|  | nCRT |  | 0.168 |
| Shrinkage SA mean (mm) | nCT | **0.012** | **< 0.001** |
|  | nCRT |  | **0.030** |
| Restaging SA mean (mm) | nCT | **0.024** | **0.012** |
|  | nCRT |  | 0.707 |
| Response rate (%)  (Restaging SA < 5 mm) | nCT | 0.067 | **0.007** |
|  | nCRT |  | 0.411 |

*The bold type indicates that the P value is statistically significant.*
