# Supplementary material for: Management of Clinically Involved Lateral Lymph Node Metastasis in Locally Advanced Rectal Cancer: A Radiation Dose Escalation Study
Source: Front Oncol. 2021 Jul 16;11:674253. doi: 10.3389/fonc.2021.674253 (PMC8322741; doi:10.3389/fonc.2021.674253)
Supplement: Supplementary file 5 [file Table_3.docx]

**SUPPLEMENTARY Table 3**. Patient clinicopathological characteristics according to the restaging LLNs (n = 202).

| **Variables** | **Restaging LLN**  **SA ≥ 5 mm** **No. (%)**  **n = 82** | **Restaging LLN**  **SA < 5 mm No. (%)**  **n = 120** | ***P*-value** |
| --- | --- | --- | --- |
| ypT stage^a^ |  |  | **0.008** |
| yp T0 | 13 (15.9) | 38 (31.7) |  |
| yp T1 | 9 (11.0) | 10 (8.3) |  |
| yp T2 | 10 (12.2) | 27 (22.5) |  |
| yp T3 | 47 (57.3) | 42 (35.0) |  |
| yp T4 | 3 (3.6) | 3 (2.5) |  |
| yp N stage^a^ |  |  | < **0.001** |
| yp N0 | 50 (61.0) | 103 (85.8) |  |
| yp N1-2 | 32 (39.0) | 17 (14.2) |  |
| AJCC/CAP TRG |  |  | **0.049** |
| 0 | 13 (15.9) | 38 (31.7) |  |
| 1 | 16 (19.5) | 22 (18.3) |  |
| 2 | 38 (46.3) | 48 (40.0) |  |
| 3 | 15 (18.3) | 12 (10.0) |  |
| Vascular invasion |  |  | 0.163 |
| negative | 79 (96.3) | 117 (97.5) |  |
| positive | 3 (3.7) | 3 (2.5) |  |
| Neural invasion |  |  | 0.969 |
| negative | 78 (95.1) | 114 (95.0) |  |
| positive | 4 (4.9) | 6 (5.0) |  |
| Surgical margin |  |  | / |
| negative | 82 (100.0) | 120 (100.0) |  |
| positive | 0 (0.0) | 0 (0.0) |  |
| Circumferential resection margin, mm |  |  | 0.225 |
| ≤ 1 | 81 (98.8) | 120 (100.0) |  |
| > 1 | 1 (1.2) | 0 (0.0) |  |
| Adjuvant chemotherapy |  |  | **0.003** |
| Yes | 78 (95.1) | 97 (80.8) |  |
| No | 4 (4.9) | 23 (19.2) |  |

*^a^yp stage is pathological stage after neoadjuvant treatment and surgical resection.*

*The bold type indicates that the P value is statistically significant.*
