# Supplementary material for: Management of Clinically Involved Lateral Lymph Node Metastasis in Locally Advanced Rectal Cancer: A Radiation Dose Escalation Study
Source: Front Oncol. 2021 Jul 16;11:674253. doi: 10.3389/fonc.2021.674253 (PMC8322741; doi:10.3389/fonc.2021.674253)
Supplement: Supplementary file 6 [file Table_4.docx]

**Supplementary Table 4.** Radiation side effects (grades 3–4) in patients who had LLNs metastasis in the nCRT and nCRT-boost subgroups

| Side effect  (Grades 3-4) | nCRT No. (%)  n = 60 | nCRT-boost No. (%)  n = 48 | *P*-value |
| --- | --- | --- | --- |
| Radiation enteritis (%) | 5 (8.3) | 5 (10.4) | 0.970 |
| Radiodermatitis (%) | 2 (3.3) | 4 (8.3) | 0.481 |
| Myelosuppression (%) | 3 (5.0) | 5 (10.4) | 0.485 |
| Total (%) | 10 (16.6) | 14 (29.1) | 0.121 |
