# Supplementary material for: Management of Clinically Involved Lateral Lymph Node Metastasis in Locally Advanced Rectal Cancer: A Radiation Dose Escalation Study
Source: Front Oncol. 2021 Jul 16;11:674253. doi: 10.3389/fonc.2021.674253 (PMC8322741; doi:10.3389/fonc.2021.674253)
Supplement: Supplementary file 7 [file Table_5.docx]

**SUPPLEMENTARY TABLE 5.** Univariate analyses of risk of local recurrence (LR), lateral local recurrence (LLR), distant recurrence (DR), and cancer specific survival (CSS) in patients with LLNs metastasis (n = 202).

| **Variable** | **3-year LR** | | | **3-year LLR** | | | **3-year DR** | | | **3-year CSS** | | |
| --- | --- | --- | --- | --- | --- | --- | --- | --- | --- | --- | --- | --- |
|  | **HR^a^** | **95% CI^b^** | ***P* value** | **HR** | **95% CI** | ***P* value** | **HR** | **95% CI** | ***P* value** | **HR** | **95% CI** | ***P* value** |
| Age (years) |  |  | 0.092 |  |  | 0.087 |  |  | 0.829 |  |  | 0.233 |
| < 55 vs. ≥ 55 | 0.557 | 0.281-1.101 |  | 0.537 | 0.263-1.095 |  | 1.079 | 0.540-2.156 |  | 0.460 | 0.128-1.649 |  |
| Sex |  |  | **0.012** |  |  | **0.013** |  |  | 0.831 |  |  | 0.840 |
| Males *vs.* females | 2.149 | 1.183-3.904 | - | 2.194 | 1.184-4.068 |  | 0.920 | 0.430-1.972 |  | 1.127 | 0.353-3.596 |  |
| Clinical T stage |  |  | 0.335 |  |  | 0.422 |  |  | 0.843 |  |  | 0.900 |
| cT2 | 1 |  |  | 1 |  |  | 1 |  |  | 1 |  |  |
| cT3 | 1.411 | 0.192-10.351 | 0.735 | 1.308 | 0.178-9.621 | 0.792 | 24684.306 | 0-5.056E+121 | 0.941 | 8268.475 | 0-2.919E+111 | 0.943 |
| cT4 | 2.247 | 0.294-17.193 | 0.435 | 2.045 | 0.266-15.739 | 0.492 | 19012.782 | 0-3.898E+121 | 0.943 | 11146.592 | 0-3.943E+111 | 0.941 |
| Clinical N stage |  |  | **0.002** |  |  | **0.001** |  |  | 0.044 |  |  | **0.030** |
| cN1 *vs*. cN2 | 3.309 | 1.537-7.124 |  | 4.293 | 1.805-10.213 |  | 2.263 | 1.023-5.005 |  | 5.272 | 1.172-23.710 |  |
| Location from anal verge (cm) |  |  | 0.448 |  |  | 0.405 |  |  | 0.750 |  |  | 0.939 |
| 0-5 *vs.* 5-10 | 1.260 | 0.694-2.289 |  | 1.300 | 0.701-2.409 |  | 0.892 | 0.441-1.802 |  | 0.958 | 0.318-2.882 |  |
| Tumor differentiation |  |  | 0.052 |  |  | 0.090 |  |  | 0.129 |  |  | 0.965 |
| Highly differentiated | 1 |  |  | 1 |  |  | 1 |  |  | 1 |  |  |
| Moderately differentiated | 1.503 | 0.696-3.249 | 0.300 | 1.365 | 0.625-2.982 | 0.434 | 2.084 | 0.836-5.193 | 0.115 | 1.151 | 0.3436-3.858 | 0.820 |
| Low differentiation | 2.762 | 1.163-6.561 | **0.021** | 2.516 | 1.042-6.077 | **0.040** | 2.945 | 1.019-8.509 | **0.046** | 1.220 | 0.221-6.726 | 0.820 |
| Neoadjuvant treatment |  |  | **< 0.001** |  |  | **0.002** |  |  | 0.565 |  |  | 0.602 |
| nCT | 1 |  |  | 1 |  |  | 1 |  |  | 1 |  |  |
| nCRT | 0.531 | 0.276-1.023 | 0.058 | 0.602 | 0.309-1.172 | 0.135 | 0.650 | 0.291-1.451 | 0.293 | 1.762 | 0.585-5.314 | 0.314 |
| nCRT-Booster | 0.062 | 0.008-0.452 | **0.006** | 0.069 | 0.009-0.507 | **0.009** | 0.802 | 0.334-1.924 | 0.622 | 0.000 | 0-2.253E+255 | 0.969 |
| Restaging MRI LLNs SA (mm) |  |  | **<0.001** |  |  | **<0.001** |  |  | **<0.001** |  |  | **<0.001** |
| < 5 *vs*. ≥ 5 | 12.158 | 5.129-28.818 |  | 13.776 | 5.394-35.180 |  | 3.149 | 1.556-6.371 |  | 13.589 | 2.975-62.065 |  |
| yp T stage^c^ |  |  | **0.018** |  |  | **0.002** |  |  | **0.001** |  |  | **0.024** |
| ypT0-2 *vs*.y pT3-4 | 3.127 | 1.636-5.978 |  | 2.800 | 1.450-5.407 |  | 3.636 | 1.695-7.791 |  | 4.361 | 1.215-15.655 |  |
| yp N stage^c^ |  |  | **<0.001** |  |  | **<0.001** |  |  | **< 0.001** |  |  | **< 0.001** |
| yp N0 | 1 |  |  | 1 |  |  | 1 |  |  | 1 |  |  |
| yp N1 | 4.833 | 2.468-9.464 | **<0.001** | 4.124 | 2.045-8.313 | **<0.001** | 3.284 | 2.045-8.313 | **0.004** | 10.578 | 3.106-36.028 | **< 0.001** |
| yp N2 | 5.236 | 2.374-11.549 | **<0.001** | 4.595 | 2.015-10.480 | **<0.001** | 6.826 | 2.922-15.575 | **< 0.001** | 6.800 | 1.603-28.853 | **0.009** |
| AJCC/CAP TRG |  |  | **0.002** |  |  | **0.006** |  |  | **0.013** |  |  | 0.415 |
| 0 | 1 |  |  | 1 |  |  | 1 |  |  | 1 |  |  |
| 1 | 4.783 | 1.294-17.682 | **0.019** | 4.740 | 1.282-17.523 | **0.020** | 4.539 | 0.915-22.509 | 0.064 | 6.188 | 0.691-55.404 | 0.103 |
| 2 | 5.344 | 1.598-17.870 | **0.006** | 5.059 | 1.508-16.975 | **0.009** | 8.511 | 2.004-36.142 | **0.004** | 5.517 | 0.677-44.927 | 0.110 |
| 3 | 8.055 | 2.214-29.309 | **0.002** | 6.308 | 1.671-23.812 | **0.007** | 3.233 | 0.540-19.365 | 0.199 | 4.525 | 0.409-50.057 | 0.218 |
| Vascular invasion |  |  | **0.001** |  |  | **0.001** |  |  | **< 0.001** |  |  | 0.104 |
| Negative *vs.* positive | 5.649 | 1.987-16.061 |  | 5.932 | 2.079-16.928 |  | 7.700 | 2.651-22.368 |  | 5.690 | 0.700-46.282 |  |
| Neural invasion |  |  | 0.132 |  |  | 0.357 |  |  | **0.004** |  |  | 0.067 |
| Negative *vs*. positive | 2.205 | 0.788-6.172 |  | 1.739 | 0.536-5.642 |  | 4.112 | 1.586-10.657 |  | 4.107 | 0.907-18.602 |  |
| Circumferential resection margin, mm |  |  | **0.007** |  |  | **0.007** |  |  | **0.013** |  |  | **0.007** |
| ≤ 1 *vs.* > 1 | 16.329 | 2.123-125.582 |  | 16.329 | 2.123-125.582 |  | 12.912 | 1.706-97.751 |  | 17.310 | 2.153-139.154 |  |
| Adjuvant chemotherapy |  |  | 0.191 |  |  | 0.240 |  |  | 0.454 |  |  | 0.641 |
| No *vs.* Yes | 2.186 | 0.677-7.060 |  | 2.022 | 0.624-6.553 |  | 1.573 | 0.481-5.146 |  | 0.698 | 0.154-3.159 |  |

*^a^HR, hazard ratio; ^b^95% CI, 95% confidence interval.*

*^c^yp stage is pathological stage after neoadjuvant treatment and surgical resection.*

*The bold type indicates that the P value is statistically significant.*
