# Supplementary material for: Management of Clinically Involved Lateral Lymph Node Metastasis in Locally Advanced Rectal Cancer: A Radiation Dose Escalation Study
Source: Front Oncol. 2021 Jul 16;11:674253. doi: 10.3389/fonc.2021.674253 (PMC8322741; doi:10.3389/fonc.2021.674253)
Supplement: Supplementary file 8 [file Table_6.docx]

**SUPPLEMENTARY TABLE 6.** Multivariate analyses of risk of local recurrence (LR), lateral local recurrence (LLR), distant recurrence (DR), and cancer-specific survival (CSS) in patients with LLNs metastasis (n = 202).

| Variable | 3-year LR | | | 3-year LLR | | | 3-year DR | | | 3-year CSS | | |
| --- | --- | --- | --- | --- | --- | --- | --- | --- | --- | --- | --- | --- |
|  | HR^a^ | 95% CI^b^ | *P* value | HR | 95% CI | *P* value | HR | 95% CI | *P* value | HR | 95% CI | *P* value |
| Age (years) |  |  |  |  |  |  |  |  |  |  |  |  |
| < 55 *vs.* ≥ 55 |  |  |  |  |  |  |  |  |  |  |  |  |
| Sex |  |  | 0.089 |  |  | **0.008** |  |  |  |  |  |  |
| Males *vs.* females |  |  |  | 2.469 | 1.271-4.799 |  |  |  |  |  |  |  |
| Clinical T stage |  |  |  |  |  |  |  |  |  |  |  |  |
| cT2 |  |  |  |  |  |  |  |  |  |  |  |  |
| cT3 |  |  |  |  |  |  |  |  |  |  |  |  |
| cT4 |  |  |  |  |  |  |  |  |  |  |  |  |
| Clinical N stage |  |  | 0.280 |  |  | 0.053 |  |  | 0.880 |  |  | 0.595 |
| cN1 *vs*. cN2 |  |  |  |  |  |  |  |  |  |  |  |  |
| Location from anal verge (cm) |  |  |  |  |  |  |  |  |  |  |  |  |
| 0-5 *vs.* 5-10 |  |  |  |  |  |  |  |  |  |  |  |  |
| Tumor differentiation |  |  |  |  |  |  |  |  |  |  |  |  |
| Highly differentiated |  |  |  |  |  |  |  |  |  |  |  |  |
| Moderately differentiated |  |  |  |  |  |  |  |  |  |  |  |  |
| Low differentiation |  |  |  |  |  |  |  |  |  |  |  |  |
| Neoadjuvant treatment |  |  | **< 0.001** |  |  | 0.058 |  |  |  |  |  |  |
| nCT | 1 |  |  |  |  |  |  |  |  |  |  |  |
| nCRT | 1.031 | 0.514-2.069 | 0.931 |  |  |  |  |  |  |  |  |  |
| nCRT-Booster | 0.075 | 0.010-0.552 | **0.011** |  |  |  |  |  |  |  |  |  |
| Restaging MRI LLNs SA (mm) |  |  | **< 0.001** |  |  | **< 0.001** |  |  | **0.048** |  |  | **0.008** |
| < 5 *vs.* ≥ 5 | 8.880 | 3.660-21.544 |  | 11.992 | 4.679-30.731 |  | 2.118 | 1.006-4.460 |  | 8.456 | 1.766-40.495 |  |
| yp T stage^c^ |  |  | 0.503 |  |  | **0.031** |  |  | 0.383 |  |  | 0.562 |
| ypT0-2 vs. ypT3-4 |  |  |  | 2.157 | 1.073-4.334 |  |  |  |  |  |  |  |
| yp N stage^c^ |  |  | **< 0.001** |  |  | 0.157 |  |  | **0.034** |  |  | **0.020** |
| yp N0 | 1 |  |  |  |  |  | 1 |  |  | 1 |  |  |
| yp N1 | 3.331 | 1.647-6.737 | **< 0.001** |  |  |  | 2.2993 | 0.958-5.516 | 0.062 | 5.170 | 1.475-18.121 | **0.010** |
| yp N2 | 4.543 | 1.956-10.549 | **< 0.001** |  |  |  | 3.562 | 1.295-9.796 | **0.014** | 4.470 | 1.031-19.377 | **0.045** |
| AJCC/CAP TRG |  |  | 0.465 |  |  | 0.390 |  |  | **0.009** |  |  |  |
| 0 |  |  |  |  |  |  | 1 |  |  |  |  |  |
| 1 |  |  |  |  |  |  | 2.944 | 0.567-15.295 | 0.199 |  |  |  |
| 2 |  |  |  |  |  |  | 5.227 | 1.191-22.947 | **0.028** |  |  |  |
| 3 |  |  |  |  |  |  | 1.360 | 0.214-8.653 | 0.745 |  |  |  |
| Vascular invasion |  |  | 0.120 |  |  | **0.001** |  |  |  |  |  |  |
| Negative *vs.* positive |  |  |  | 6.890 | 2.263-20.982 |  |  |  |  |  |  |  |
| Neural invasion |  |  |  |  |  |  |  |  | 0.167 |  |  |  |
| Negative *vs*. positive |  |  |  |  |  |  |  |  |  |  |  |  |
| Circumferential resection margin, mm |  |  | 0.352 |  |  | 0.820 |  |  |  |  |  | 0.186 |
| ≤ 1 *vs*. > 1 |  |  |  |  |  |  |  |  |  |  |  |  |
| Adjuvant chemotherapy |  |  |  |  |  |  |  |  |  |  |  |  |
| No *vs.* Yes |  |  |  |  |  |  |  |  |  |  |  |  |

*^a^HR, hazard ratio; ^b^95% CI, 95% confidence interval.*

*^c^yp stage is pathological stage after neoadjuvant treatment and surgical resection.*

*The bold type indicates that the P value is statistically significant.*
